# Supplementary material for: CANTO-RT: One of the Largest Prospective Multicenter Cohort of Early Breast Cancer Patients Treated with Radiotherapy including Full DICOM RT Data
Source: Cancers (Basel). 2023 Jan 25;15(3):751. doi: 10.3390/cancers15030751 (PMC9913384; doi:10.3390/cancers15030751)
Supplement: Supplementary file 1 [file cancers-15-00751-s001.zip › cancers-2150763-supplementary.pdf]

**Table S1:** List of main variable.

| Variable name | Variable Label | assigned variable names | Variable Format |
|---|---|---|---|
| Age at enrolment | Age in year | Age_diag | numeric |
| Smoking status at diagnosis | 1=Current; 2=Former ; 3=Never | TABAC_BI | character |
| Diabetes | 0=No; 1=Yes; | Diabete_atcd | character |
| Hypertension | 0=No; 1=Yes; | HTA_atcd | character |
| Dyslipidemia | 0=No; 1=Yes; | dyslipidemie_atcd | character |
| BMI | kg/m2 | IMC_BI | numeric |
| Tumour size (pT) | 0=T0; 1=T1; 2=T2; 3=T3 | PTGlobal | character |
| Nodal status (pN) | 0=0; 1=1; 3=2; 4=3 | PN_M0 | character |
| Tumour histology | 1=Infiltrating Ductal; 2=Lobular; 3=Others (including mixed). | Tumour_histo | character |
| Hormone Receptors positive | 0=Negative; 1=Positive; | HR | character |
| Estrogen receptors | 0=Negative; 1=Positive; | RE | character |
| Progesterone receptors | 0=Negative; 1=Positive; | RP | character |
| HER2 | 0=Negative; 1=Positive; | HER2 | character |
| SBR Grading | 1=I; 2=II; 3=III. | GRADE | character |
| Ki67 | 0=No; 1=Yes; | Ki67 | character |
| Percent of Ki67 | % | POURCKI67 | numeric |
| Type of chemotherapy | 0=No chemotherapy; 1=Neo-adjuvant chemotherapy; 2=Adjuvant chemotherapy; 3=Peri-adjuvant chemotherapy (neo + adjuvant); | CHIMIOTYP | character |
| Hormonal therapy | 0=No; 1=Yes; | Tumour_histo | character |
| Trastuzumab treatment | 0=No; 1=Yes; | Base_herceptin | character |
| Lumpectomy | 0=No; 1=Yes; | TUMOR_ | character |
| Total mastectomy | 0=No; 1=Yes; | MASTR_ | character |
| Sentinel node | 0=No; 1=Yes; | GANGLS_ | character |
| Axillary dissection | 0=No; 1=Yes; | CURAGE_ | character |

| | | | |
|---|---|---|---|
| Radiation therapy | 0=No; 1=Yes; | RADIO_ | character |
| Patients with boost | 0=No; 1=Yes; | Boost_ | character |
| Lymph node levels treated | 0=None; 1=Yes; | Aires_GG_ | character |
| Level 1 | 0=None; 1=Yes; | I_all_ | character |
| Level 2 | 0=None; 1=Yes; | II_all_ | character |
| Level 3 | 0=None; 1=Yes; | III_all_ | character |
| Level 4 | 0=None; 1=Yes; | IV_all_ | character |
| Internal mammary chain | 0=None; 1=Yes; | CMI_all_ | character |
| Irradiation techniques | 0=3D; 1=IMRT | Technique_ | character |
| Fractionation regimens | 0=Normofractionation 25-fractions; 1=Hypofractionation 15-16 fractions; 2=Hypofractionation and Partial breast irradiation; | Fractionnement__NF0__HF1_ | character |
